# *Centella asiatica* Attenuates Diabetes Induced Hippocampal Changes in Experimental Diabetic Rats

**DOI:** 10.1155/2014/592062

**Published:** 2014-08-05

**Authors:** Nelli Giribabu, Nelli Srinivasarao, Somesula Swapna Rekha, Sekaran Muniandy, Naguib Salleh

**Affiliations:** ^1^Department of Physiology, Faculty of Medicine, University of Malaya, 50603 Lembah Pantai, Kuala Lumpur, Malaysia; ^2^Department of Materials Science and Engineering, National Chiao Tung University, University Road, Hsinchu 300, Taiwan; ^3^Department of Zoology, Sri Venkateswara University, Tirupati, Andhra Pradesh 517502, India; ^4^Department of Molecular Medicine, Faculty of Medicine, University of Malaya, 50603 Lembah Pantai, Kuala Lumpur, Malaysia

## Abstract

Diabetes mellitus has been reported to affect functions of the hippocampus. We hypothesized that *Centella asiatica*, a herb traditionally being used to improve memory, prevents diabetes-related hippocampal dysfunction. Therefore, the aim of this study was to investigate the protective role of *C. asiatica* on the hippocampus in diabetes. *Methods*. Streptozotocin- (STZ-) induced adult male diabetic rats received 100 and 200 mg/kg/day body weight (b.w) *C. asiatica* leaf aqueous extract for four consecutive weeks. Following sacrifice, hippocampus was removed and hippocampal tissue homogenates were analyzed for Na^+^/K^+^-, Ca^2+^- and Mg^2+^-ATPases activity levels. Levels of the markers of inflammation (tumor necrosis factor, TNF-*α*; interleukin, IL-6; and interleukin, IL-1*β*) and oxidative stress (lipid peroxidation product: LPO, superoxide dismutase: SOD, catalase: CAT, and glutathione peroxidase: GPx) were determined. The hippocampal sections were visualized for histopathological changes. *Results*. Administration of *C. asiatica* leaf aqueous extract to diabetic rats maintained near normal ATPases activity levels and prevents the increase in the levels of inflammatory and oxidative stress markers in the hippocampus. Lesser signs of histopathological changes were observed in the hippocampus of *C. asiatica* leaf aqueous extract treated diabetic rats. *Conclusions*. *C. asiatica* leaf protects the hippocampus against diabetes-induced dysfunction which could help to preserve memory in this condition.

## 1. Introduction

The hippocampus is an area of the brain that is involved in short- [1] and long-term [2] memory. In rats, amnesia can be caused by hippocampal dysfunction [3], whereas, in higher animals including primates, memory loss could occur due to dysfunctions of both hippocampus and amygdala [4]. The loss of neurons or axonal degeneration secondary to ischemia can result in deficits of the hippocampus-dependent spatial memory [5]. Mild to moderate traumatic brain injury was capable of producing prolonged spatial memory deficit in rats without evidence of neuronal death [3]. Diabetes has been linked to memory impairment in rats [6] and humans [7]. On the other hand, hypoglycemia secondary to insulin overdose can also cause hippocampal injury which could result in anterograde amnesia [8].

Hyperglycemia could induce oxidative stress in the hippocampus [9] resulting in apoptosis of hippocampal synapses and neurons [10]. Hippocampal oxidative stress is associated with increased level of lipid peroxidation products [11] and diminution of activity levels of endogenous antioxidant enzymes [12]. Diabetes has been found to inhibit activity of hippocampal Na^+^-K^+^-ATPase or Na^+^ pump [13]. Diabetes has also been reported to suppress activity of Mg^2+^-ATPase [14] and Ca^2+^-ATPase in rat whole brain [15]. The effect on Mg^2+^-ATPase and Ca^2+^-ATPase activity in the hippocampus of diabetic rats however remains unknown.

*Centella asiatica*, a herb from Mackinlayoideae family, is native to the wetlands in the tropical and subtropical regions of Asia.* C. asiatica* has been widely used in Ayurvedic, African, and Chinese traditional medicine [16]. This herb was found to promote wound healing via stimulating cellular proliferation and collagen synthesis at the wound site [17], strengthening gastric mucosal barrier, and preventing ethanol-induced gastritis [18].* C. asiatica* has also been shown to modulate the immune system [19], prevent blood coagulation [20], act as antioxidant [21], prevent alleviation of oxidative stress [22], act as anti-inflammatory agent [23], and inhibit proliferation of cancer cells [24]. Yet, the most widely reported health benefit of this herb is in improving the brain function, particularly related to memory and learning. In fact,* C. asiatica* has been reported to stimulate nerve regeneration* in vitro* [25] which could explain its brain protective effect. Administration of this herb to juvenile and young adult mice or rats was found to enhance their learning and memory performances [26, 27]. Meanwhile, in humans,* C. asiatica* has been reported to improve mental ability of the mentally retarded children [28] and could selectively decrease the amyloid-*β* levels in the hippocampus [29].

We hypothesized that* C. asiatica* was effective in protecting the hippocampus against oxidative stress and inflammation caused by diabetes. This study therefore investigated the effect of* C. asiatica* leaves on hippocampal function as reflected by the levels of ATPases activity. Additionally, evidence of alleviation of hippocampal destruction as indicated by decreased levels of oxidative stress and inflammation and lesser signs of histopathological changes in diabetic rats was also sought.

## 2. Material and Methods 

### 2.1. Chemicals

Streptozotocin (STZ) was purchased from Sigma-Aldrich Company (St. Louis, MO, USA). All other chemicals were of analytical grade.

### 2.2. Preparation of Leaf Aqueous Extract of* C. asiatica*

*C. asiatica* leaves were collected from Tirumala Hills, Andhra Pradesh, India. The taxonomic identification of the plant material was confirmed by the botanist at the Department of Botany, Sri Venkateswara University, Tirupati, Andhra Pradesh, India. The leaves were dried in the shade and crushed into fine powder. The aqueous extract was prepared by soaking 1 kg of powder in 3 L of distilled water for 48 hrs. The extracted material was filtered through Whatman number 1 (0.45 *μ*m Ref. HAWP04700, Bedford, MA, USA) filter paper. This process was repeated three or four times until the extract was rendered colorless. The extract was distilled and concentrated under reduced pressure in rotary evaporator (Rotavapor, R-210, Buchi Labortechnik, AG, Flawil, Switzerland) at 50 ± 5°C and lyophilized using freeze-dryer (Telstar, Barcelona, Spain), which yielded 7% (w/w) of freeze-dried material.

### 2.3. Phytochemical Screening

The freshly prepared aqueous leaf extract of* C. asiatica* was qualitatively tested for the presence of chemical constituents. Phytochemical screening of the extract was performed by using standard procedures [30] and phytochemical constituents were identified based on the characteristic color changes.

### 2.4. Experimental Animals

Adult male Wistar rats (12 weeks old, weighing 180 ± 10 g) were purchased from Animal House, Faculty of Medicine, University of Malaya, Kuala Lumpur, Malaysia. The animals were maintained under standard laboratory conditions (temperature 22 ± 1°C; 12 h light/dark cycle) and had free access to water and commercial pellet diet (Harlan diet, UK). Experimental procedures were in accordance with ARRIVE guidelines (Animals in Research: Reporting* In Vivo* Experiments) and European Community Guidelines/EEC Directive, 1986. This study was approved by the Animal Care and Use Committee, Faculty of Medicine, University of Malaya, with ethics number 2013-07-15/FIS/R/NS.

### 2.5. Acute Toxicity Studies

Acute toxicity studies were conducted according to the guidelines by the Organization for Economic Cooperation and Development (OECD, 2001). The extract was dissolved in distilled water with 1% sodium carboxymethyl cellulose (Na-CMC) at 2 mL/kg and was administered to experimental animals by using oral gavage tube. Fifty male Wistar rats were divided into five groups with each group receiving a single dose of 100, 250, 500, 1000, and 2000 mg/kg b.w of* C. asiatica* leaf aqueous extract. Animals were continuously monitored for 4 hrs for behavioral (alertness, restlessness, irritability, vomiting, and fearfulness), neurological (spontaneous activity, convulsion, gait, bleeding orifices, and touch/pain response), and autonomic (defecation, micturition) changes. The number of demised rats in each group was recorded after 24–72 hrs. The extract was found to have no toxic effects when administered in doses up to 2000 mg/kg b.w. Hence, in this study, 100 and 200 mg/kg b.w of this extract were used in accordance with the previously reported doses which were safe [31].

### 2.6. Induction of Diabetes in Experimental Animals

Overnight-fasted adult male Wistar rats were rendered diabetic via a single intraperitoneal (i.p) injection of STZ (55 mg/kg) dissolved in 0.1 M cold citrate buffer (pH 4.5) [32]. The rats were allowed to drink 5% glucose solution overnight to overcome drug-induced hypoglycemia. Rats in control group received citrate buffer (i.p). Fasting blood glucose (FBG) levels were measured 3 days after STZ injection and only animals with FBG levels between 300 and 400 mg/kg/day were selected for the experiment. Treatment with the extract was started on the third day following STZ injection which was considered day one.* C. asiatica* leaf aqueous extract was administered in a form of suspension orally by using gavage tube daily for 28 consecutive days.

### 2.7. Experimental Design

A total of 30 rats were used and were divided into five experimental groups of six rats per group.

 Group  I: normal nondiabetic rats receiving vehicle (Na^+^-CMC suspension) only.
 Group  II: nontreated diabetic rats receiving vehicle (Na^+^-CMC suspension) only.
 Group  III: diabetic rats treated with* C. asiatica* leaf aqueous extract at 100 mg/kg b.w.
 Group  IV: diabetic rats treated with* C. asiatica* leaf aqueous extract at 200 mg/kg b.w.
 Group V: diabetic rats treated with standard antidiabetic agent (glibenclamide) at 600 *μ*g/kg b.w.

### 2.8. Collection of Blood and Hippocampus

28 days after the starting of treatment, rats were weighed and overnight-fasted rats were sacrificed with i.p injection of pentobarbitone sodium (60 mg/kg b.w) anesthesia followed by cervical dislocation. Blood was collected from each rat via intracardiac puncture and allowed to clot for 30 min at room temperature. The serum was separated by centrifugation (Thermo Scientific, Model 75005286, USA) at 3000 rpm for 15 minutes. The right hippocampus was immediately harvested, washed with ice-cold saline, and weighed prior to preparation of tissue homogenate. The left hippocampus was used for histological study. The tissue somatic index was determined by the following formula:

(1) $$\begin{array}{c} \frac{\text{weight}\;\text{of}\;\text{the}\;\text{tissue}\;\text{in}\;\text{grams}}{\text{weight}\;\text{of}\;\text{body}\;\text{in}\;\text{grams}}\times 100. \end{array}$$

### 2.9. Histopathological Study

The left hippocampus was fixed with 10% neutral paraformaldehyde, dehydrated through ascending concentrations of ethyl alcohol, cleared in xylene, embedded in paraffin, and then cut manually using a microtome (Histo-Line Laboratories, ARM-3600, Via Brembo, Milan, Italy) to obtain 5 *μ*m thick sections. The sections were deparaffinized and rehydrated through descending concentrations of ethyl alcohol and stained with hematoxylin and eosin (H&E). The stained tissues were dehydrated in 80% alcohol followed by 95% ethyl alcohol, placed in two changes of 100% ethyl alcohol, and cleansed with two changes of xylene. Histopathological examinations were carried out by using a phase contrast microscope with an attached camera (Nikon H600L, Tokyo, Japan).

### 2.10. Preparation of Tissue Homogenate

The excised right hippocampus was homogenized in ice-cold 50 mM sodium phosphate buffer (pH 7.4) containing 0.1 mM ethylenediaminetetraacetic acid (EDTA) by using a glass-teflon homogenizer (Heidolph Silent Crusher M, Germany). The supernatant was separated by means of centrifugation at 1000 ×g for 20 min at 4°C and was then frozen at −80°C until use for biochemical analysis.

### 2.11. Determination of Lipid Peroxidation (LPO) and Antioxidant Enzyme Activities

The malondialdehyde (MDA) content, a measure of LPO, was assayed in the form of thiobarbituric acid-reactive substances (TBARS) according to the method of Esterbauer and Cheeseman [33]. The rate of lipid peroxidation was expressed as *μ* moles of MDA formed/gram wet weight of tissue. SOD (EC 1.15.1.1) activity of the homogenates was assayed according to the method of Misra and Fridovich [34]. The assay procedure involves inhibition of epinephrine autoxidation in alkaline medium (pH 10.2) to adrenochrome, in the presence of this enzyme. SOD activity level was expressed as the amount of enzyme that inhibits oxidation of epinephrine by 50%, which was equal to 1U per milligram of protein. CAT (EC 1.11.1.6) activity was determined by decomposition of H_2_O_2_ at 240 nm for 3 min monitored spectrophotometrically [35]. The activity of this enzyme was expressed in *μ*mol of hydrogen peroxide (H_2_O_2_) metabolized/mg protein/min. GPx (EC 1.11.1.9) activity level was determined according to the method by Rotruck et al. [36] and was expressed as *μ*mol of GSH consumed/mg protein/min.

### 2.12. Determination of Inflammatory Markers in the Hippocampus

Tumor necrosis factor alpha (TNF-*α*) and interleukins (IL-1*β*, IL-6) were measured in the supernatant of homogenized hippocampal tissue by using ELISA kits (Biosource International Inc., Camarillo, CA). The procedures were carried out according to the manufacturer's guidelines. TNF-*α*, IL-1*β*, and IL-6 were determined from a standard curve and their levels were expressed in pg/mL.

### 2.13. Determination of ATPase Activity Levels

Na^+^/K^+^-ATPase (EC 3.6.1.3) activity was estimated in the supernatant of hippocampal tissue homogenates according to the method of Bonting [37]. The activity of calcium-dependent ATPase (Ca^2+^-ATPase) was assayed according to the method of Hjertén and Pan [38]. Meanwhile, the activity of magnesium-dependent ATPase (Mg^2+^-ATPase) was assayed according to the method of Ohnishi et al. [39]. Enzymes activities were expressed in *μ*mol pi/min/mg protein. The protein concentration in hippocampal homogenate was determined according to the method of Lowry et al. [40].

### 2.14. Statistical Analyses

All data were expressed as mean ± S.D. of six determinants. Statistical significance was evaluated by one-way analysis of variance (ANOVA) using SPSS version 7.5 (SPSS, Cary, NC, USA) and individual comparisons were made by Duncan's multiple range test (DMRT).

## 3. Results 

### 3.1. Phytochemical Screening of Aqueous Leaf Extract of* C. asiatica*

Preliminary phytochemical screening revealed the presence of alkaloids, flavonoids, glycosides, saponins, terpenoids, steroids, linens, phenols, and tannins (data not shown).

### 3.2. Effect of Aqueous Leaf Extract of* C. asiatica* on FBG Levels and Tissue Somatic Index of the Hippocampus

The effects of aqueous extract of* C. asiatica* leaves on FBG levels at days 0 and 28 and tissue somatic index of the hippocampus in rats from the different treatment groups are shown in Table 1. In nontreated diabetic rats, the FBG levels were significantly (*P* < 0.001) higher than normal, nondiabetic rats. However, diabetic rats treated with 100 and 200 mg/kg of* C. asiatica* leaf aqueous extract showed significantly (*P* < 0.01) lower FBG levels on day 28 as compared to nontreated diabetic rats. In diabetic rats, hippocampal tissue somatic index was not statistically different from normal, nondiabetic rats (Table 1).

### 3.3. Effect of Aqueous Extract of* C. asiatica* Leaves on MDA and Antioxidant Enzymes Activity Levels in the Hippocampus

**Figure 1:**
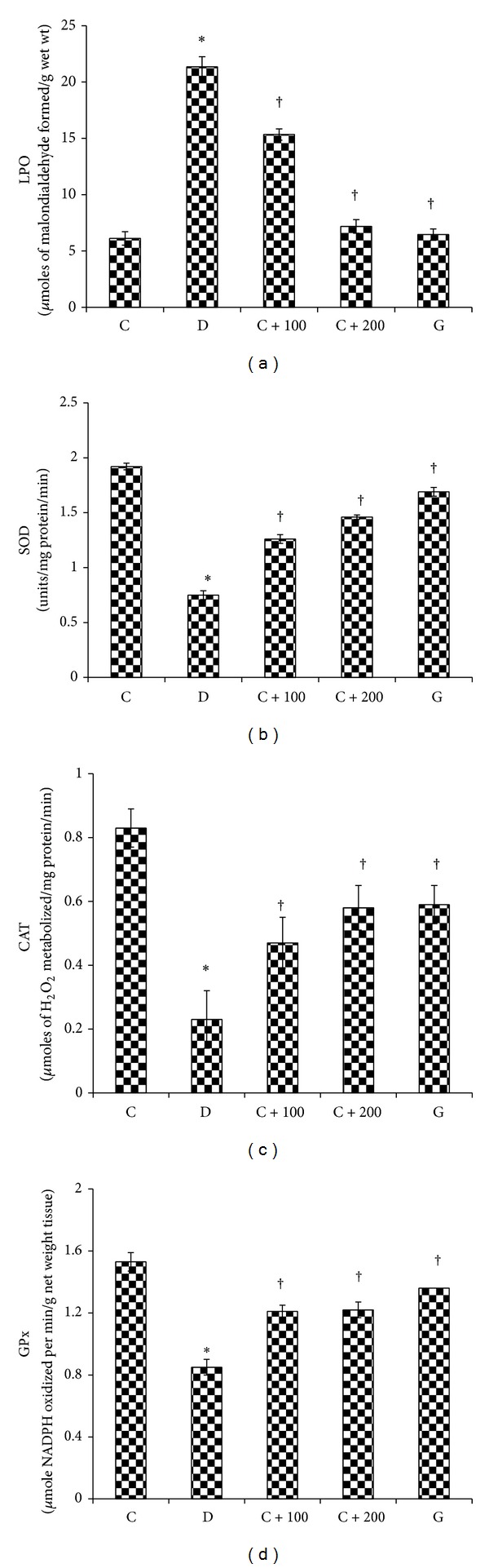
The levels of lipid peroxidation (LPO) product and antioxidant enzymes (SOD, CAT, and GPx) in hippocampal homogenates. LPO level was the highest in diabetic rats. Meanwhile, the levels of SOD, CAT, and GPx were the lowest in diabetic rat hippocampus. Treatment with* C. asiatica* leaf extract reversed changes in these parameters in diabetic rat hippocampus. *P < 0.05 as compared to normal, nondiabetic rats; ^†^ *P* < 0.05 as compared to nontreated diabetic rats.

MDA levels were significantly (*P* < 0.01) higher in the hippocampal homogenates of nontreated diabetic rats as compared to normal, nondiabetic rats (Figure 1). 28-day treatment of diabetic rats with 100 and 200 mg/kg b.w* C. asiatica* leaf aqueous extracts or glibenclamide resulted in a significantly (*P* < 0.01) lower MDA level in the hippocampal homogenates as compared to nontreated diabetic rats.

SOD activity was 60.93% lower in STZ-induced diabetic rats' hippocampus as compared to normal, nondiabetic rats. However, treatment of diabetic rats with 100 and 200 mg/kg b.w* C. asiatica* leaf aqueous extract or glibenclamide resulted in a significantly higher SOD activity in the hippocampal homogenate. CAT activity was significantly (*P* < 0.01) lower in nontreated diabetic rats as compared to normal, nondiabetic rats. Treatment of diabetic rats with* C. asiatica* leaf aqueous extract or glibenclamide resulted in a significantly higher CAT activity level as compared to nontreated diabetic rats (Figure 1). In nontreated diabetic rats, GPx activity in the hippocampus was significantly (*P* < 0.01) lower than that in normal, nondiabetic rats. Treatment of diabetic rats with 100 and 200 mg/kg b.w* C. asiatica* leaf aqueous extracts or glibenclamide resulted in a significantly higher GPx activity level in the hippocampus as compared to nontreated diabetic rats (Figure 1).

### 3.4. Effect of* C. asiatica* Leaf Extract on the Hippocampal Inflammatory Markers

**Figure 2:**
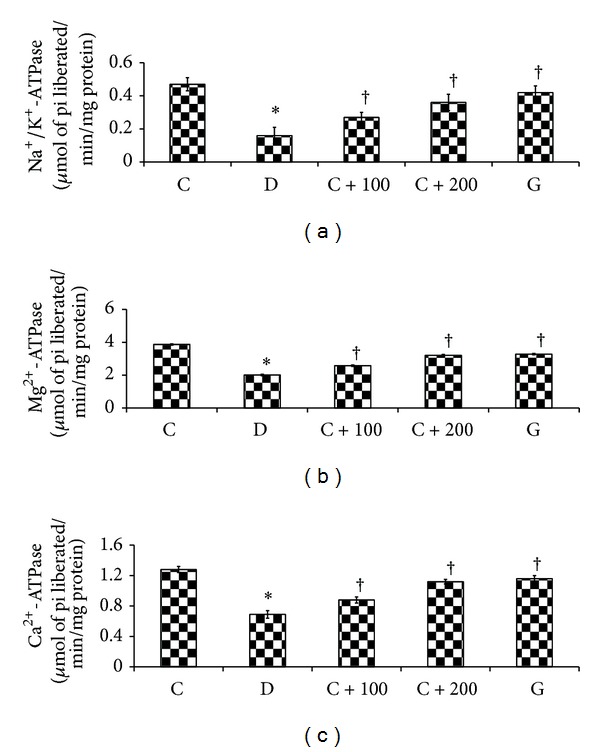
Activity levels of Na^+^/K^+^-ATPase, Mg^2+^-ATPase, and Ca^2+^-ATPase in hippocampal homogenates. ATPases activity levels were the lowest in diabetic rats. Treatment with* C. asiatica* leaf extract reversed changes in ATPases activity levels in diabetic rats. *P < 0.05 as compared to normal, nondiabetic rats; ^†^ *P* < 0.05 as compared to nontreated diabetic rats.

The effect of* C. asiatica* leaf aqueous extract on inflammatory markers in the hippocampus of rats in different experimental groups is shown in Figure 2. In nontreated diabetic rats, the levels of TNF-*α* in the hippocampal homogenates were 252.38% higher than normal, nondiabetic rats. Diabetic rats treated with* C. asiatica* leaf aqueous extract at doses of 100 and 200 mg/kg b.w had a significantly lower TNF-*α* level in the hippocampal homogenates as compared to nontreated diabetic rats. A significantly higher IL-1*β* (306.31%) was observed in the hippocampus of nontreated diabetic rats as compared to normal, nondiabetic rats. Treatment of diabetic rats with 100 and 200 mg/kg b.w* C. asiatica* leaf aqueous extract resulted in a significantly lower levels of IL-1*β* in the hippocampus as compared to nontreated diabetic rats (*P* < 0.01). The hippocampal IL-6 levels were significantly higher in nontreated diabetic rats as compared to normal, nondiabetic rats (*P* < 0.01). Diabetic rats treated with 100 and 200 mg/kg b.w* C. asiatica* leaf aqueous extract had a significantly lower IL-6 level in the hippocampus as compared to nontreated diabetic rats (*P* < 0.01).

### 3.5. Effect of* C. asiatica* Leaf Extracts on the Hippocampal ATPase Activity Levels

**Figure 3:**
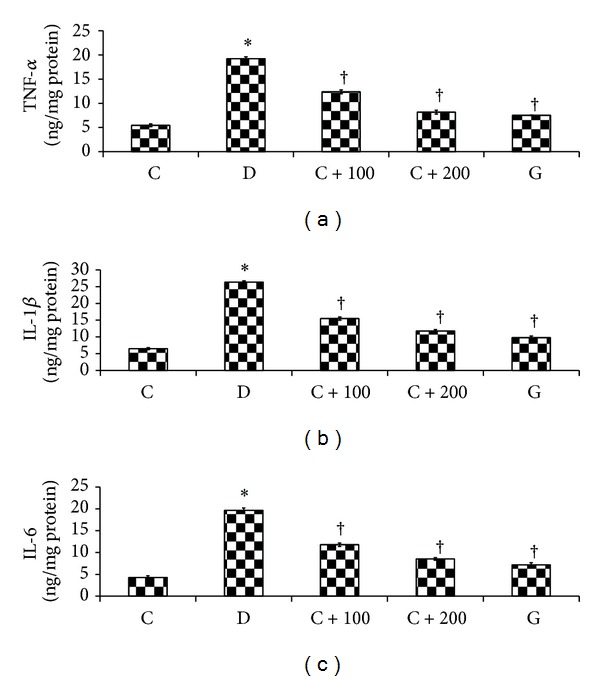
The levels of TNF-*α*, IL-1*β*, and IL-6 in hippocampal homogenates. The levels of inflammatory markers were the highest in diabetic rats. Treatment with* C. asiatica* leaf extract reversed changes of inflammatory markers levels in hippocampal homogenates of diabetic rats. *P < 0.05 as compared to normal, nondiabetic rats; ^†^ *P* < 0.05 as compared to nontreated diabetic rats.

Figure 3 shows the effect of* C. asiatica* leaf aqueous extract on ATPases activity of the hippocampus of rats in different experimental groups. The Na^+^/K^+^-ATPase activity was 65.95% lower in the hippocampus of nontreated diabetic rats as compared to normal, nondiabetic rats. Treatment of diabetic rats with 100 and 200 mg/kg b.w* C. asiatica* leaf aqueous extract or glibenclamide resulted in a significantly higher hippocampal Na^+^/K^+^-ATPase activity as compared to nontreated diabetic rats (*P* < 0.01). Mg^2+^-ATPase activity was significantly lower (48.06%) in nontreated diabetic rats' hippocampus as compared to normal, nondiabetic rats. Diabetic rats treated with* C. asiatica* leaf aqueous extract at 100 and 200 mg/kg b.w or glibenclamide showed a significantly higher hippocampal Mg^2+^-ATPase activity as compared to nontreated diabetic rats (*P* < 0.01). Meanwhile, Ca^2+^-ATPase activity in the hippocampus of diabetic rats was significantly lower than normal, nondiabetic rats (*P* < 0.01). Ca^2+^-ATPase activity in the hippocampus of diabetic rats treated with* C. asiatica* leaf aqueous extract at 100 and 200 mg/kg b.w or glibenclamide was significantly higher than nontreated diabetic rats (*P* < 0.01).

### 3.6. Histopathological Changes of the Hippocampus following Treatment with* C. asiatica* Leaf Aqueous Extract

**Figure 4:**
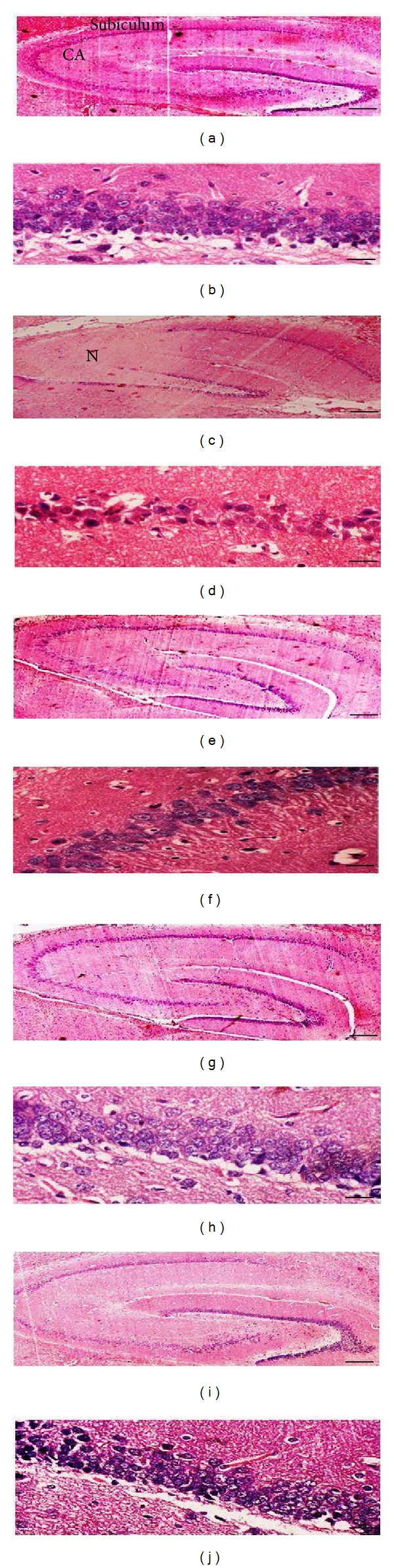
Photographs of hippocampal sections of rats in different experimental groups. There were signs of necrosis in diabetic rat hippocampus (c). Loss of neuronal and glial cells was observed in diabetic rat hippocampus (d). Administration of* C. asiatica* leaf extract or glibenclamide ((e)–(j)) preserved near normal hippocampal histology in diabetic rats. N = necrosis; CA = cornu amonis.

Figure 4 shows histological sections of the hippocampus from normal nondiabetic, nontreated diabetic, and diabetic rats treated with* C. asiatica* leaf extract or glibenclamide. Our findings showed that there were signs of necrosis in the subiculum, presubiculum, and cornu ammonis (CA) areas of the hippocampus of diabetic rats. Administration of* C. asiatica* prevented the development of necrotic changes in these areas. Higher magnification images revealed a significant decrease in the number of neurons and glial cells in the hippocampus of diabetic rats. Administration of 200 mg/kg/day b.w* C. asiatica* leaf extract resulted in higher hippocampal neurons and glial cells number as compared to nontreated diabetic rats.

## 4. Discussion

Our findings have revealed the protective effect of* C. asiatica*, a herb widely consumed by Asians, against hippocampal destruction due to oxidative stress in diabetes.* C. asiatica* has been used in Ayurvedic medicine to improve the memory and learning abilities [41]. Administration of this herb during postnatal period in mice has been shown to enhance learning and memory [26]. Studies in humans have indicated that* C. asiatica* could improve memory in mentally retarded children [28]. In addition,* C. asiatica* combined with other herbal extracts has been shown to improve memory in patients with Alzheimer's disease [42]. These effects were attributed to the ability of this herb to preserve normal hippocampal function. We have provided evidence which showed that consumption of* C. asiatica* could help preserve memory function in diabetes, known to be associated with long- and short-term memory impairment [43]. Diabetes has been found to cause degenerative changes in the hippocampus [10] which contributed towards memory loss. Our histopathological findings including neuronal degeneration in diabetic rat hippocampus were consistent with the previous reports which indicate similar changes in the hippocampus under this condition [9, 44, 45].

Our findings showed that hippocampal ATPases activities were reduced in diabetes. Hippocampal Na^+^/K^+^-ATPase has been linked to memory function in rodents [46]. Hippocampal neurons [47] and interneurons express Na^+^/K^+^-ATPase in their axonal and dendritic membranes [48]. Insulin has been reported to stimulate activity of this pump in the rat hippocampus [49]. In conditions associated with lack of insulin such as diabetes, hippocampal Na^+^/K^+^-ATPase activity was reduced, indicating that insulin is essential for maintaining the normal function of this pump [50]. A marked decrease in Na^+^/K^+^-ATPase activity could adversely affect hippocampal function. Critical role of this pump in normal hippocampal function was evidence from impairment of hippocampal neuron activity in disease associated with mutation of this protein [51]. Besides hyperglycemia, hippocampal Na^+^/K^+^-ATPase activity was also inhibited by amino acids such as homocysteine [52] and drugs such as ouabain [53] and in ischaemia [54]. In our study, the decrease in activity of this pump in diabetes could be prevented by the administration of* C. asiatica* leaves, suggesting ability of this herb to prevent hyperglycemia-induced inhibition on the Na^+^/K^+^-ATPase activity.

The expression of Ca^2+^-ATPase pump has been reported in rodent [55] and human [56] hippocampus. In this study, we have shown that hippocampal Ca^2+^-ATPase activity was markedly reduced in diabetes. Besides diabetes, activity of this pump was also found to be reduced in seizure [57]. The expression of neuron-specific endoplasmic reticulum Mg^2+^-ATPase was reported in the hippocampal neurons [58]. Our study has shown that activity of Mg^2+^-ATPase pump was reduced in diabetic condition. Treatment with* C. asiatica* leaf extract in diabetic rats could help preserve both Ca^2+^- and Mg^2+^-ATPases activities of the hippocampus, therefore restoring normal hippocampal function. In view that activity of all three major hippocampal ATPases was conserved,* C. asiatica* leaves might be useful in preserving and enhancing memory in diabetes.

We have shown that the levels of LPO product in the hippocampus of diabetic rats were increased indicating oxidative stress. Oxidative stress has been linked to neurodegeneration [59]. Oxidative stress in the hippocampus could be increased by guanylic acid [60] and could be prevented by melatonin [61]. Homocysteine was also reported to induce hippocampal oxidative stress [62]. In diabetes, increased formation of reactive oxygen species (ROS) in the hippocampus could cause oxidative stress resulting in neuronal apoptosis [63]. We have shown that activity of hippocampal antioxidant enzymes such as SOD, CAT, and GPx was decreased in diabetes which could contribute towards oxidative stress. A similar observation has been reported in homocystinuria where increased hippocampal oxidative stress was caused by decreased activity of endogenous antioxidant enzymes [62]. Antioxidant enzymes help to scavenge free radicals such as nitric oxide, superoxide, and peroxides, levels of which were markedly elevated in diabetes [59]. In our study, ability of* C. asiatica* to reduce hippocampal oxidative stress may be attributed to the free radical scavenging effect and the ability of this herb to preserve near normal activity levels of endogenous antioxidant enzymes.

Our findings have indicated that there were signs of hippocampal inflammation in diabetic rats from the histopathological changes and elevation of inflammatory markers (IL-1*β*, IL-6, and TNF-*α*) in hippocampal homogenates. IL-6 was reported to produce detrimental effect on the hippocampus [64]. Higher levels of interleukins (ILs) could activate the central inflammatory mechanisms that result in hippocampal neurodegeneration leading to memory impairment. An inverse correlation between peripheral IL-6 levels and memory has been reported in human adults during the midlife period [65], suggesting that IL levels could affect memory. We have shown that administration of* C. asiatica* leaf extract resulted in the levels of hippocampal inflammatory markers in diabetic rats to be reduced to near normal, which indicates decreased hippocampal inflammation. Meanwhile, normalization of FBG levels following administration of* C. asiatica* leaf might also contribute to the reduction in hippocampal inflammation in diabetes.

Evidence of necrotic changes was observed in cornu ammonis (CA) and subiculum areas of the hippocampus in diabetic rats with severe loss of pyramidal neurons and glial cells (Figure 4). A previous study reported that in diabetes, neuronal degeneration was due to apoptosis [66]. These changes were parallel with the reported decrease in hippocampal neurogenesis in mice with diabetes [67]. Administration of* C. asiatica* leaf extract to diabetic rats prevented neuronal degeneration and therefore helps in maintaining normal hippocampal function in diabetes. In addition to* C. asiatica*, we have shown that antidiabetic drug glibenclamide could also prevent hippocampal neuronal degeneration in diabetes.

In conclusion, our study has provided mechanisms which could explain the protective role of* C. asiatica* leaf against hippocampal dysfunction which include preservation of near normal activity levels of the major hippocampal ATPases and the reduction in inflammatory changes and oxidative stress in the hippocampus in diabetes. Cumulatively, these effects justify the claims that* C. asiatica* contributes towards the preservation of memory function in diabetes.

## Figures and Tables

**Table 1:** Effect of *C. asiatica *leaf aqueous extract on FBG levels and hippocampal tissue somatic index in experimental diabetic rats.

Parameters	Normal	Diabetic	Diabetic		
			100 mg/kg b.w *C. asiatica *	200 mg/kg b.w *C. asiatica *	600 *μ*g/kg b.w glibenclamide
0th day blood glucose levels	94.38 ± 7.27	438.27∗ ± 9.83	436.81^ns^ ± 7.69	437.68^ns^ ± 9.63	436.65^ns^ ± 9.76
28th day blood glucose levels	98.64 ± 8.67	432.16∗ ± 7.16	217.37^†^ ± 8.36	203.44^†^ ± 8.42	182.16^†^ ± 8.71
Hippocampal tissue somatic index	0.04 ± 0.01	0.04^ns^ ± 0.01	0.04^ns^ ± 0.01	0.04^ns^ ± 0.01	0.04^ns^ ± 0.01

Value represents means ± S.D. for 6 rats per group. ∗*P* < 0.01 as compared to normal, nondiabetic rats group; ^†^ *P* < 0.01 as compared to nontreated diabetic rats.
